# Ferroptosis is involved in focal segmental glomerulosclerosis in rats

**DOI:** 10.1038/s41598-023-49697-8

**Published:** 2023-12-14

**Authors:** Yue Shi, Xiujie Shi, Mingming Zhao, Yifan Zhang, Qi Zhang, Jing Liu, Hangyu Duan, Bin Yang, Yu Zhang

**Affiliations:** 1 Department of Nephrology, Xiyuan Hospital, China Academy of Chinese Medical Sciences, No. 1, Xiyuan Playground, Haidian District, Beijing, 100091 China; 2 Department of Pathology, Xiyuan Hospital, China Academy of Chinese Medical Sciences, No. 1, Xiyuan Playground, Haidian District, Beijing, 100091 China

**Keywords:** Diseases, Medical research, Nephrology

## Abstract

To explore whether ferroptosis is involved in focal segmental glomerulosclerosis (FSGS) and its mechanism. The FSGS rat model was constructed by single nephrectomy combined with fractional tail vein injection of doxorubicin. 24-hour urine protein, serum biochemistry, HE, PAS and Masson pathological staining were measured to assess renal injury. Glomerular and morphological changes of ferroptosis were observed by transmission electron microscopy. Iron content in renal tissue was assessed by Prussian blue staining and iron detection. GSH/GSSG kit was used to detect the content and proportion of reduced/oxidized glutathione. Lipid peroxidation related proteins including MDA expression was assessed by colorimetry. The iron metabolism biomarkers such as hepcidin, ferroportin and TFR, ferroptosis biomarkers such as GPX4, ACSL4, and ferritinophagy biomarkers such as LC3II/LC3I, NCOA4, and FTH1 were detected by Western blot. Significant urinary protein, hyperlipidemia, azotemia, increased serum creatinine and hypoproteinemia were observed in FSGS rats. Histology and electron microscopy showed segmental sclerosis of glomeruli, compensatory enlargement of some glomeruli, occlusion of capillary lumen, balloon adhesion, increased mesangial matrix, atrophy of some tubules, and renal interstitial fibrosis in renal tissue of FSGS rats. The morphology of glomerular foot processes disappeared; the foot processes were extensively fused and some foot processes detached. Mitochondria became smaller, membrane density increased, and mitochondrial cristae decreased or disappeared. In addition, iron deposition was observed in renal tissue of FSGS rats. Compared with the control group, the levels of GSH, GSH/GSSG, GPX4, and ferroportin were reduced and the expression of GSSG, MDA, ACSL4, hepcidin, and TFR was increased in the renal tissue of FSGS rats; meanwhile, the expression of LC3II/LC3I and NCOA4 was increased and the expression of FTH1 was decreased. Ferroptosis is involved in the pathological progression of FSGS, which is probably associated with activation of ferritinophagy. This represents a potential therapeutic target for FSGS.

## Introduction

Focal segmental glomerulosclerosis (FSGS) is a pathomorphological diagnosis first defined by Rich in 1957, characterized by sclerosis of the affected glomerular segments, with tubular atrophy and renal interstitial fibrosis^1^. Clinically, FSGS is characterized by massive proteinuria and nephrotic syndrome, with a tendency of chronic progression. Approximately 50% of FSGS patients progress to end-stage renal disease (ESRD) within 5–10 years and are major contributors to ESRD^2^. In the past 20 years, the incidence of FSGS has been increasing year by year worldwide^3^. Although numerous studies have made some significant achievements in the etiology and pathogenesis of FSGS, including genetic susceptibility, circulating serum soluble urokinase receptor, viral infection, hyperlipidemia and lipid peroxidation, and podocyte injury^4–7^, the pathogenesis of FSGS is currently inconclusive. Recently, scholars have focused on podocyte injury. Studies have shown that cell death such as apoptosis and autophagy is involved in the process of podocyte injury in FSGS^8,9^.

Ferroptosis is a novel mode of cell death, first defined by Dixon in 2012, with iron accumulation and lipid peroxidation as the main biochemical features^10^. Lipid reactive oxygen species (ROS), including hydrogen peroxide, undergo the Fenton reaction catalyzed by ferrous iron and oxidize polyunsaturated fatty acids (PUFAs) on lipid membranes, which produces excessive lipid peroxides and disrupts the integrity of lipid membranes, thereby triggering the development of ferroptosis. In recent years, much evidence has confirmed that ferroptosis involves the pathogenesis of various diseases, such as neurological diseases, malignant tumors, and cardiovascular diseases^11–13^. In the field of renal diseases, the close relationship between ferroptosis and acute kidney injury (AKI), chronic kidney disease (CKD), and diabetic nephropathy (DKD) has attracted much attention^14–16^. Recently, He et al.^17^ found that CPX4 and GSH were decreased in FSGS podocytes and tubular epithelial cells, and ferroptosis inhibitor could improve the damage of FSGS podocytes, suggesting that ferroptosis was involved in the occurrence of FSGS. However, their simplification of ferroptosis was limited to CPX4 and GSH and did not describe the iron metabolic disorders and mechanisms underlying ferroptosis in FSGS. At present, the iron metabolism and mechanism underlying ferroptosis in FSGS remains largely unknown.

Ferroptosis involves complicated regulatory mechanisms, mainly including antioxidant pathways, lipid metabolism pathways, and iron metabolism pathways. Glutathione (GSH) and glutathione peroxidase 4 (GPX4) are major contributors to the lipid repair system with strong antioxidant capacity^18^. Acyl-CoA synthetase long chain family member 4 (ACSL4) and malondialdehyde (MDA) are key markers of lipid peroxidation, and their up-regulation can promote ferroptosis^19^. Ferroptosis is an iron-dependent process in which iron metabolism plays a critical role. Iron metabolism involves the absorption, storage, utilization, and efflux of iron^20^. Ferritin Heavy Chain 1 (FTH1) is the main component of iron storage protein, which is the substrate for ferritinophagy. Nuclear receptor coactivator 4 (NCOA4) is a selective cargo receptor that binds to FTH1 and delivers it to lysosomes for autophagic degradation, thereby supplementing the abundance of intracellular iron during iron deficiency, further inducing the Fenton response and ferroptosis in a process known as ferritinophagy^21^. Deng et al.^22^ found that in cisplatin-induced AKI cell model, ferritinophagy mediated the release of free iron, producing massive lipid peroxidation and triggering ferroptosis in HK-2 cells. Ferritinophagy may be an important mechanism inducing ferroptosis^23^. To date, the involvement and mechanism of ferroptosis in FSGS are unknown.

Therefore, we established a rat model of FSGS to investigate the roles of ferroptosis in the pathological process of FSGS and its possible mechanism by detecting iron overload, lipid peroxidation, and the levels of ferroptosis and ferritinophagy-related regulatory proteins in the renal tissue of FSGS rats.

## Materials and methods

### Animals

The study was carried out in accordance with the ARRIVE guidelines and national animal experiment guidelines and were approved by the Ethics Committee of Xiyuan Hospital (Ethical Approval No. 2022XLA088-1). 12 SPF SD male rats, weighing 180 ± 220 g, were provided by Beijing Vital River Corporation. They were kept in SPF animal room of Xiyuan Hospital, China Academy of Chinese Medical Sciences at constant temperature 25 ± 2 °C, relative humidity 40–70%, light/dark cycle of 12 h/12 h. All rats were allowed to drink and eat freely.

After 1 week of adaptive feeding, rats were randomly divided into control group and FSGS model group, with 6 rats in each group. The rat model of FSGS was established by left nephrectomy combined with two tail vein injections of doxorubicin. 3% pentobarbital sodium solution was administered with abdominal anesthesia at a dose of 1.5 mL/100 g. The left renal area was fully exposed and local skin preparation was performed. The skin was incised after routine disinfection and the peripheral fat of the left kidney was dissected. Subsequently, the left renal portal vessel was ligated and the left kidney was removed. Finally, the muscular layer and incision were sutured. Benzylpenicillin sodium for injection (Sichuan Pharmaceutical Preparation Co. LTD, H51021742) 100,000 units/day was injected intramuscularly for 3 consecutive days after surgery to prevent infection. Doxorubicin (Shenzhen Main Luck Pharmaceuticals Inc., H44024359) 4 mg/kg and 2 mg/kg was injected into the tail vein at 7 and 28 days after surgery. In the control group, only the same dose of normal saline was injected into the tail vein. The rats were anesthetized with 3% pentobarbital sodium solution and blood samples were collected from the abdominal aorta after 24 h of urine collection at the end of the 12th week after surgery. And a little of renal tissue was cut into 1 mm^3^ pieces and fixed in 4% glutaraldehyde. Part of the remaining renal tissue was fixed in 4% paraformaldehyde, and the other part was cryopreserved in a  − 80 °C freezer for subsequent experiments.

### Biochemical analysis of serum and urine

Blood was collected from the abdominal aorta and centrifuged at 3000 rpm for 15 min to retain serum at the end of the experiment. Blood urea nitrogen (BUN), serum creatinine (Scr), total cholesterol (TC), triglyceride (TG) and albumin (ALB) were measured by automatic biochemical analyzer. 24-hour urine protein quantification was measured by pyrophenol red/molybdate method. Blood and urine indexes were measured at the Department of Clinical Laboratory, Xiyuan Hospital, China Academy of Chinese Medical Sciences.

### Renal histopathology

Renal tissue samples were fixed overnight in 10% neutral formalin and dehydrated in ethanol and xylene. Then they were embedded in paraffin and sectioned to 5 μm for storage on glass slides. Sections were stained with hematoxylin–eosin (HE). Subsequently, PAS and Masson trichrome staining were performed according to the manufacturer 's instructions to detect pathological changes in glomeruli, which were observed and photographed by a light microscope (DP73, OLYMPUS, Japan).

### Transmission electron microscopy

Small pieces of renal tissue were immobilized in 4% glutaraldehyde and preserved at 4 °C for 24 h. After washing with precooled PBS, they were fixed in 1% osmic acid, dehydrated in graded acetone and ethanol, and embedded in epoxy resin. Ultrathin sections (0.1 μm thick) were double-stained with uranyl acetate and lead citrate. Transmission electron microscopy was used to observe and photograph.

### Prussian blue staining

Iron deposition in renal tissue was detected using the Prussian Blue Iron Stain Kit (Solarbio, G1422). Thin sections of 5 μm were prepared as previously described and treated with dehydration and transparency. Followed by 40 min of Perls stain and thorough rinsing with distilled water for 3 min. Nuclear fast red stain was added and nuclei were lightly stained for 3 min. The sections were rinsed in tap water for 3 s. Then they underwent routine dehydration transparent and neutral resin seal, ultimately observed and photographed with a light microscope.

### GSH, GSSG, GSH/GSSG, MDA, and Fe^2+^ assays

Renal tissues were rapidly frozen with liquid nitrogen and powdered. Protein removal reagent S was added and thoroughly homogenized with a glass homogenizer, obtaining supernatant after centrifugation. GSH and GSSG in renal tissue were detected according to the instructions of the GSH/GSSG kit (Beyotime, S0053). PBS buffer was added to the renal tissue and mechanically homogenized in an ice-water bath. Then it was centrifuged to collect supernatant. MDA levels in the renal tissue were determined with reference to the instructions of the lipid oxidation (MDA) assay kit (Beyotime, S0131). Approximately 10 mg of renal tissue was washed with cold PBS, homogenized in iron detection buffer and centrifuged at 16,000 g for 10 min to obtain supernatant. Iron content in renal tissue was detected in line with the manufacturer 's instructions of the iron assay kit (abcam, ab83366).

### Western blot analysis

Renal tissues were homogenized in cold 1% PMSF lysate and centrifuged to obtain the supernatant as the resulting protein extract. Protein concentration was determined using BSA protein kit (wanleibio, WLA004). The protein lysates were separated on 8–15% SD-PAGE gels and transferred to PVDF membranes (Millipore, IPVH00010). Subsequently, they were blocked with 5% skimmed milk powder on a shaker for 1 h at room temperature. PVDF membranes were incubated overnight at 4 °C with corresponding primary antibodies, including anti-hepcidin (1:500; affinity, DF6492), anti-TFR(1:2000; abmart, T56618), anti-ferroportin (1:500; BOSTER, A01953-2), anti-ACSL4 (1:10,000; abcam, ab155282), anti-GPX4 (1:5000; abcam, ab125066), anti-FTH1 (1:500; ABclonal, A19544), anti-LC3 II (1:500; wanleibio, WL01506), and anti-NCOA4 (1:1000; ABclonal, A5695). PVDF membranes after incubation with primary antibodies were washed with TBST for 4 times and incubated with goat anti-rabbit IgG-HRP (1:5000; wanleibio, WLA023) for 45 min at room temperature. Enhanced chemiluminescence was used for protein detection and blot visualization, and gel image processing system (Gel-Pro-Analyzer software) was used to detect the relative protein density values of the target bands.

### Statistical analysis

Quantitative data were presented as mean ± standard deviation. The independent sample t test was used for comparison of two groups. The data were analysed by SPSS version 26.0 for Windows (SPSS Inc., Chicago, IL, USA). *P* < 0.05 was considered statistically significant.

## Results

### 24-hour urine protein and serum biochemical analysis results

**Figure 1:**
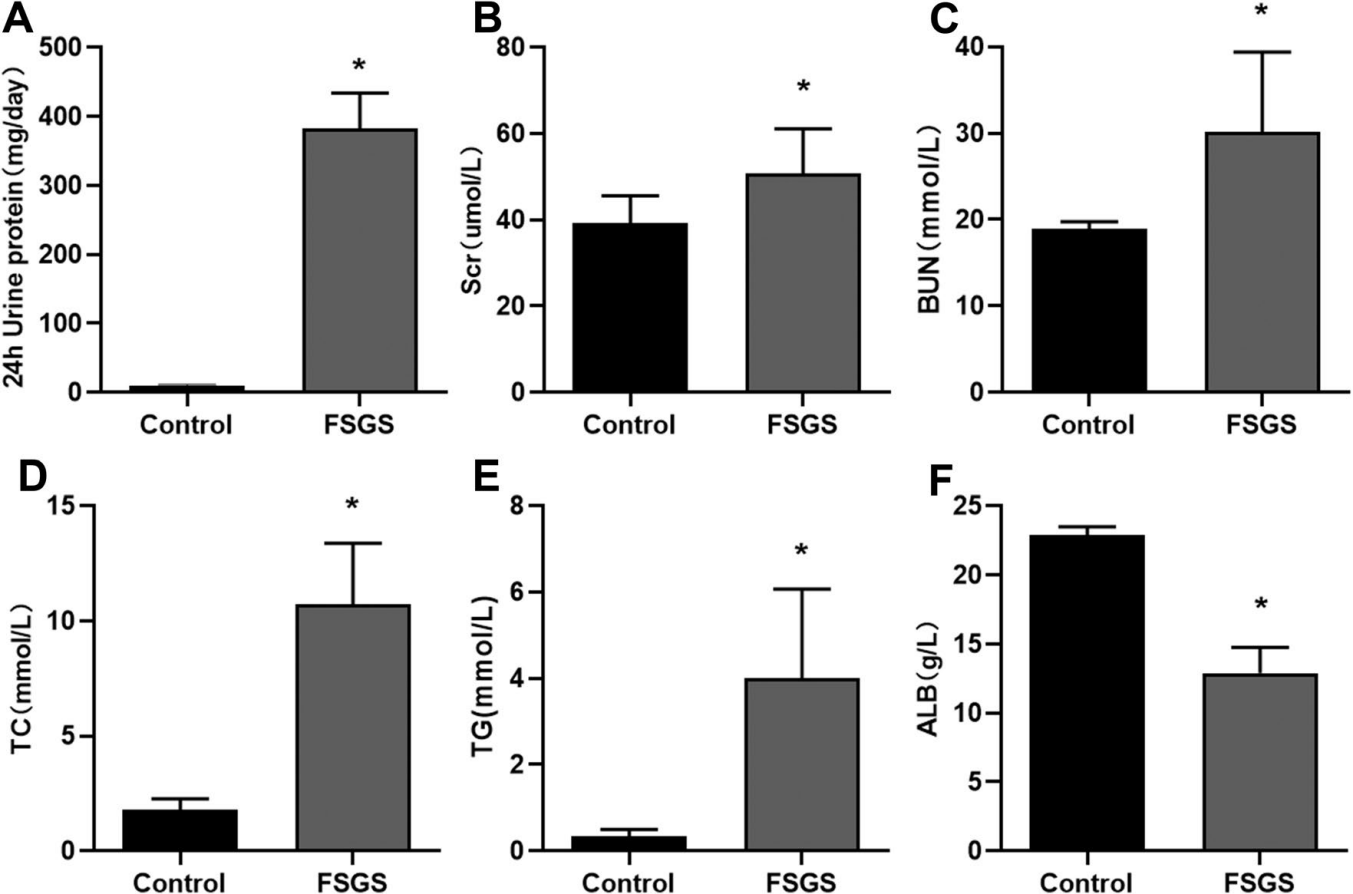
FSGS rats exhibited characteristics of nephrotic syndrome. (**A**) Quantitative results of 24-hour urine protein. (**B**) Scr results. (**C**) BUN results. (**D**) TC results. (**E**) TG results. (**F**) ALB results. *P < 0.05.

The data showed that the 24-hour urine protein quantification of FSGS rats was significantly higher than that of the control group after the end of modeling. Serum biochemical analysis revealed that the levels of Scr, BUN, TC, and TG were increased in FSGS rats compared with controls, along with a decrease in ALB (Fig. 1). These results were consistent with the clinical features of nephrotic syndrome in FSGS.

### Morphological and pathological evaluation of renal tissue

**Figure 2:**
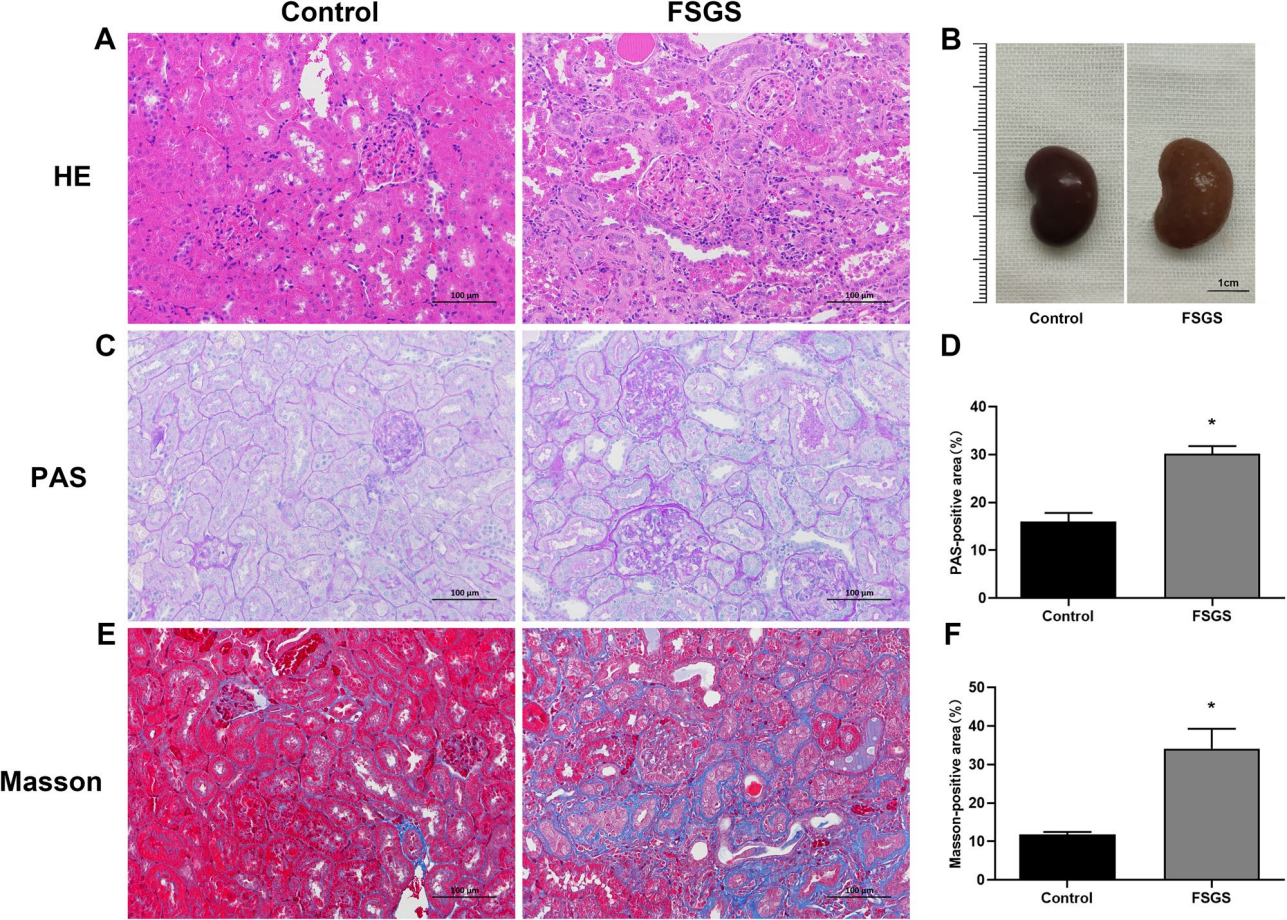
Morphological and pathological images. (**A**) HE staining (scale bar: 100 μm). (**B**) Morphological manifestations of kidney (scale bar: 1 cm). (**C**) PAS staining (scale bar: 100 μm). (**D**) PAS-positive area. (**E**) Masson staining (scale bar: 100 μm). (**F**) Masson-positive area. *P < 0.05.

Following kidney removal, we observed its morphology. Normal rat kidneys were regular in size, red in color, and smooth in surface. In FSGS rats, the kidneys were edematous, lighter in color, and rough in surface with significant granular sensation (Fig. 2). HE, PAS and Masson staining were performed on renal tissue to further observe the pathological changes and evaluate the establishment of FSGS model. The glomerular vascular loops were thin and clear in normal rats. Segmental sclerosis of glomeruli, compensatory enlargement of partial glomeruli, partial occlusion of capillary lumen, balloon adhesion, increased mesangial matrix, atrophy of some tubules, and even renal interstitial fibrosis were observed in the renal tissue of FSGS rats.

### Transmission electron microscopic observation of renal tissue

**Figure 3:**
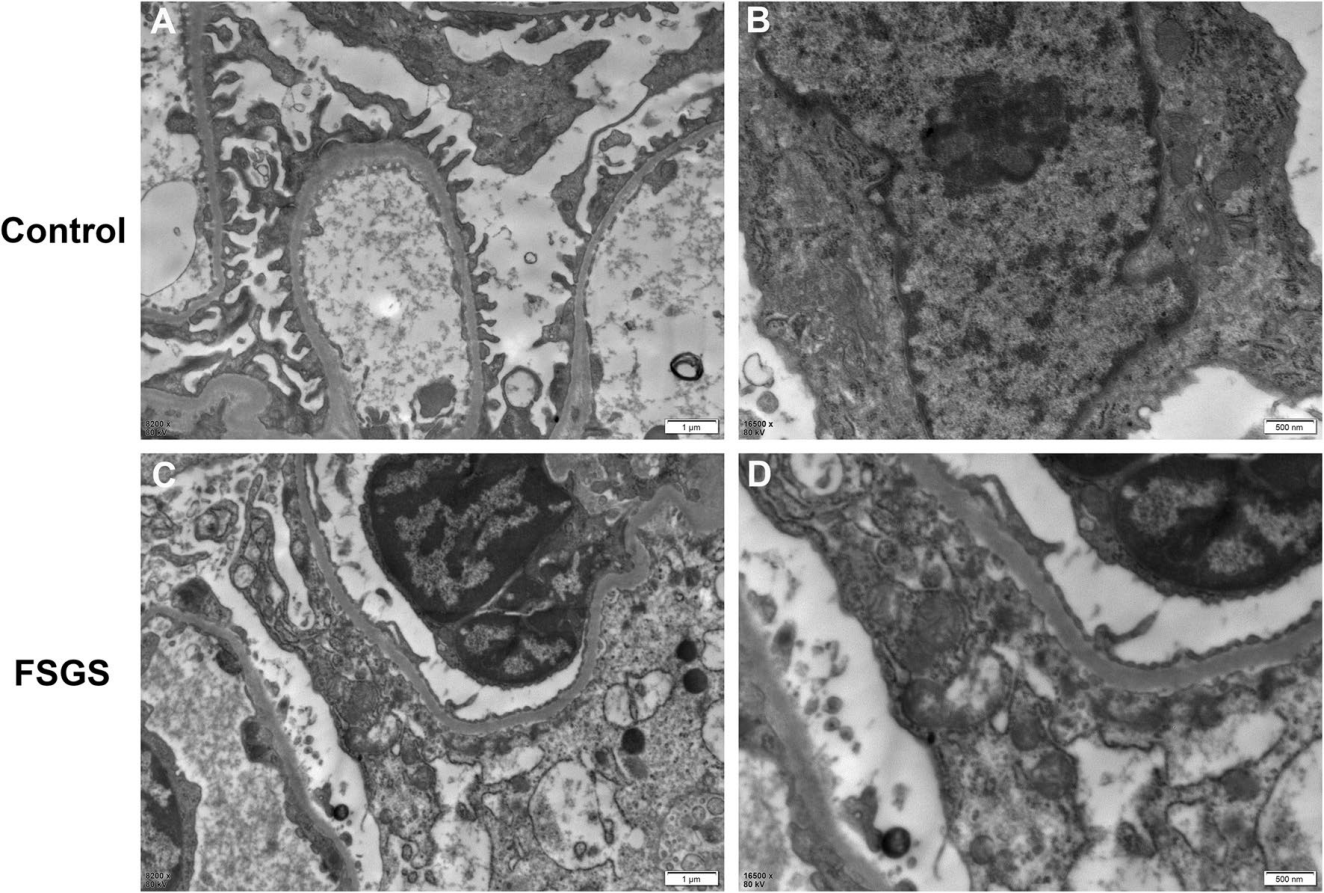
Representative transmission electron microscope images. (**A**) Normal rat podocyte foot processes (scale bar: 1 μm). (**B**) Mitochondrial morphology of normal rat podocytes (scale bar: 600 nm). (**C**) Fusion of FSGS rat podocyte foot processes (scale bar: 1 μm). (**D**) Mitochondrial morphology of FSGS rat podocytes (scale bar: 600 nm).

Transmission electron microscopy showed that the morphology of podocyte foot processes in glomeruli of rats in the normal group was regular and orderly arranged (Fig. 3). The morphology of podocyte foot processes disappeared, and the foot processes were diffusely fused in the renal tissue of FSGS rats. Partial foot processes were detached, and no electron dense deposits were observed. The ultrastructural morphology of mitochondria showed that the size of intracellular mitochondria varied and some mitochondria became smaller. In addition, the membrane of mitochondria density increased, and mitochondrial cristae decreased or disappeared, presenting ultrastructural morphological characteristics of ferroptosis.

### Iron metabolism in renal tissue

**Figure 4:**
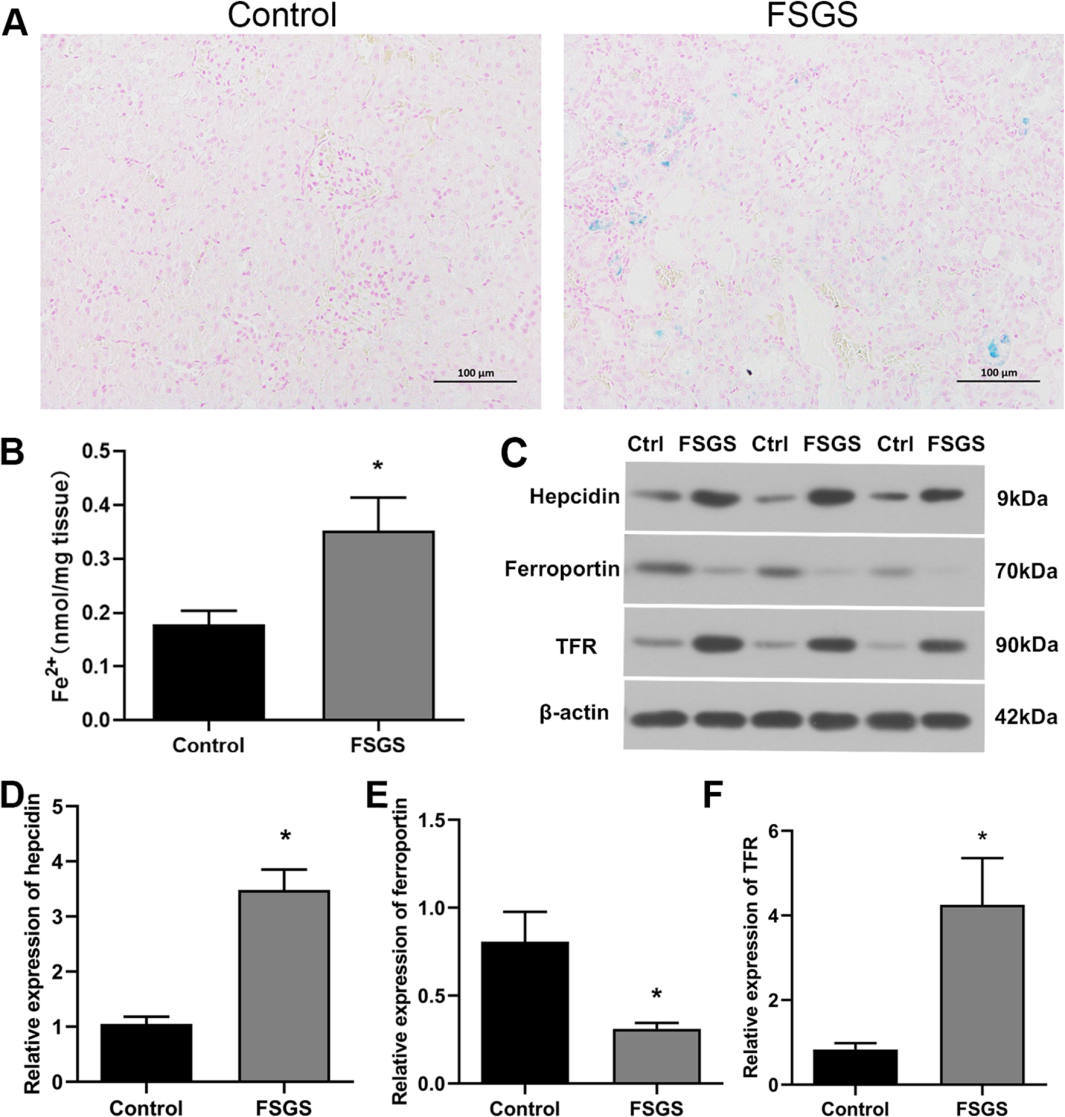
FSGS rats showed iron metabolism disorder. (**A**) Prussian blue staining (scale bar: 100 μm). (**B**) Fe^2+^ results. (**C**) Western blot analysis of hepcidin, ferroportin and TFR. (**D**) Relative expression of hepcidin. (**E**) Relative expression of ferroportin. (**F**) Relative expression of TFR. *P < 0.05.

Next, we observed iron deposition in renal tissue of rats in both groups. In general, the sites of hemosiderin deposition were blue and the nuclei and other tissues were red after Prussian blue staining. The results showed that the renal tissue of FSGS rats appeared obvious blue staining. In addition, the iron ion detection results also showed that the Fe^2+^ level in the renal tissue of FSGS rats was significantly higher than that of the control group. In addition, Western blot results showed that the expression of transferrin receptor (TFR) and hepcidin in renal tissue of FSGS rats increased, while ferroportin expression decreased (Fig. 4), indicating that there was significant iron deposition in the renal tissue of FSGS rats.

### The expression of ferroptosis-related biomarkers GSH, GSSG, GSH/GSSG, GPX4, MDA and ACSL4

**Figure 5:**
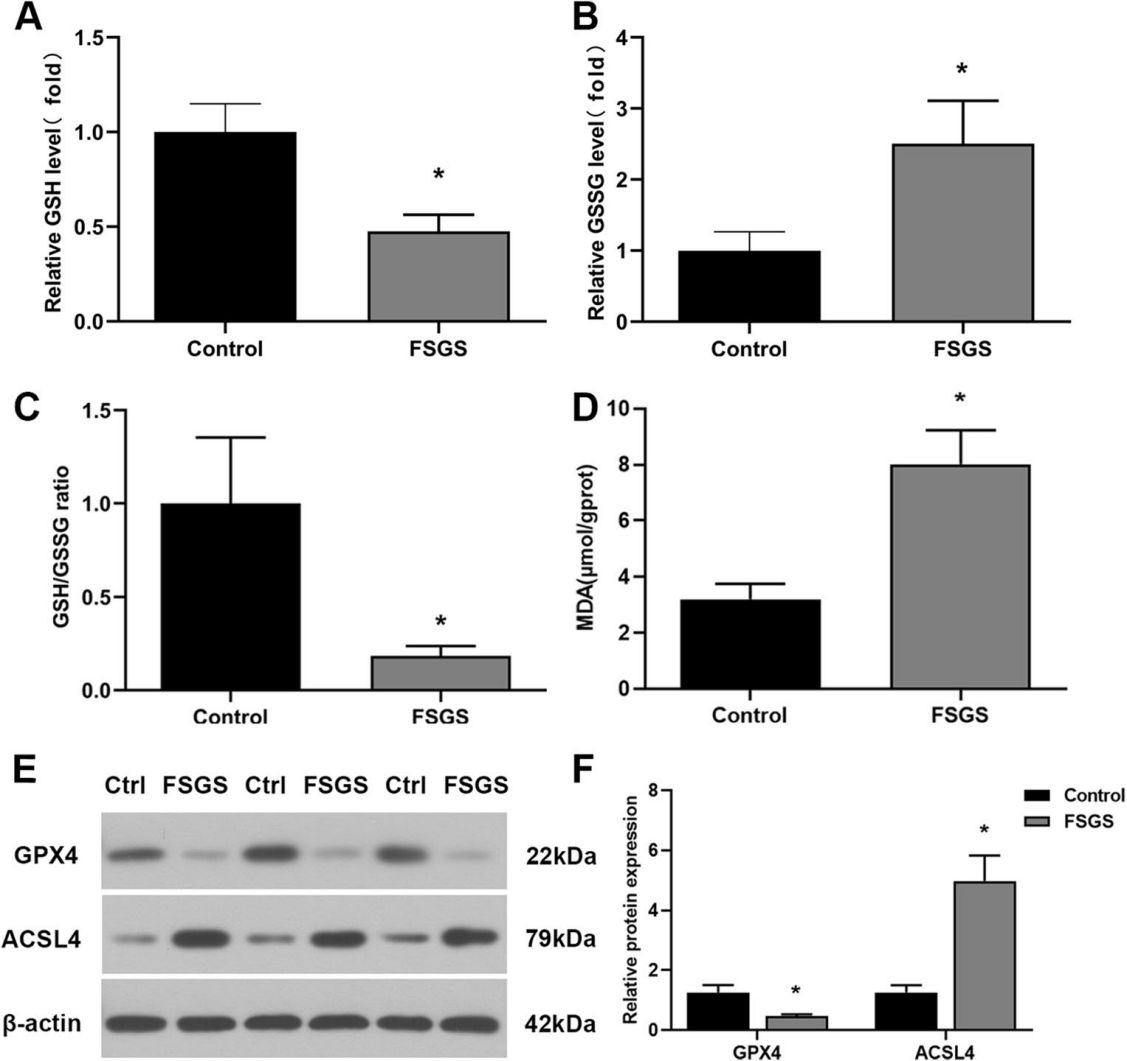
Characteristic manifestations of ferroptosis in FSGS rats. (**A**) GSH results. (**B**) GSSG results. (**C**) GSH/GSSG ratio. (**D**) MDA results. (**E**) Western blot analysis of GPX4 and ACSL4. (**F**) Relative expression of GPX4 and ACSL4. *P < 0.05.

We measured the expression of ferroptosis-related biomarkers such as GSH, GSSG, GSH/GSSG, GPX4, MDA and ACSL4 to further investigate the involvement of ferroptosis in FSGS rats. The results showed that the levels of GSH, GSH/GSSG, and GPX4 were greatly decreased, whereas the expression of GSSG, MDA and ACSL4 was increased in the renal tissue of FSGS rats (Fig. 5), suggesting that the renal tissue of FSGS rats exhibited decreased antioxidant capacity, oxidative stress and lipid peroxidation.

### The expression of ferritinophagy-related biomarkers LC3II/LC3I, FTH1, and NCOA4

**Figure 6:**
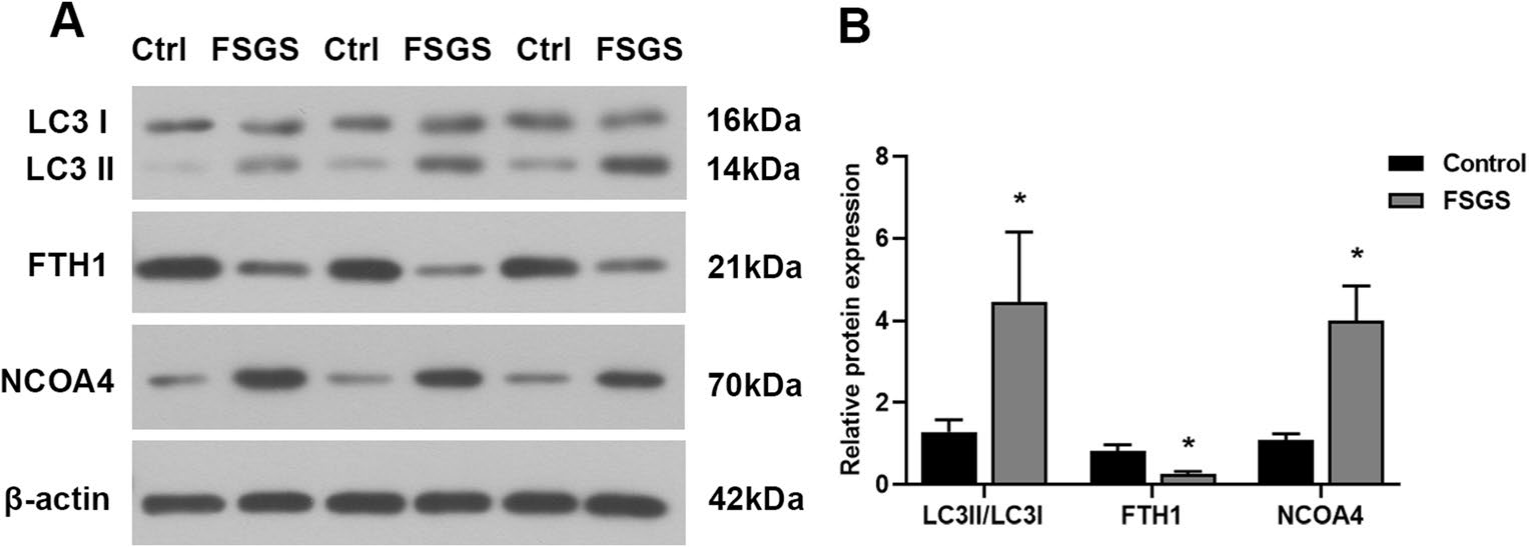
Characteristic manifestations of ferritinophagy in FSGS rats. (**A**) Western blot analysis of LC3II/LC3I, FTH1 and NCOA4. (**B**) Relative expression of LC3II/LC3I, FTH1 and NCOA4. *P < 0.05.

Given the close relationship between ferritinophagy and ferroptosis, we detected ferritinophagy-specific biomarkers, including LC3II/LC3I, FTH1, and NCOA4 to determine whether ferritinophagy is involved in the development of ferroptosis in FSGS rats. Western blot results showed that the LC3II/LC3I ratio was increased and the level of NCOA4 was increased in the renal tissue of FSGS rats, while the content of FTH1 was significantly decreased (Fig. 6), indicating that ferritinophagy was activated in the renal tissue of FSGS rats and may mediate the occurrence of ferroptosis.

## Discussion

FSGS is a common pathological type of nephrotic syndrome in children and adults. Focal and segmental glomerulosclerosis was observed in FSGS under light microscope, and significant foot process fusion of glomerular podocytes was observed under electron microscope. FSGS is an important cause of hormone resistance and ESRD, and its incidence has gradually increased in recent years; however, the pathogenesis of FSGS has not been clarified^3^. It is generally accepted that podocyte injury is a critical event in the pathogenesis of FSGS. Podocytes, a highly differentiated cell attached to the glomerular basement membrane (GBM), are the last barrier to the glomerular filtration membrane. Podocyte injury leads to podocyte loss and depletion, mesangial matrix proliferation, capillary lumen occlusion, GBM exposure, and massive urinary protein leakage, further resulting in glomerulosclerosis^24,25^. Currently, various cell death including apoptosis, autophagy, and necrosis are involved in the process of podocyte loss in FSGS^8,9^. As a novel cell death mode, the role of ferroptosis in FSGS remains unclear. Therefore, we established a rat model of FSGS to explore the role and mechanism of ferroptosis in FSGS.

Doxorubicin is a broad-spectrum anticancer agent that was reported to induce renal injury as early as 1976^26^. Doxorubicin causes podocyte injury and subsequent focal glomerulosclerosis and fibrosis, closely resembling FSGS. The doxorubicin nephropathy model used to simulate the FSGS experimental model has been recognized at home and abroad^27,28^. Therefore, we used unilateral nephrectomy combined with fractional injection of low-dose doxorubicin to establish the FSGS model. The results showed that the urinary protein content, the Scr, BUN, TC and TG levels were significantly increased, and the ALB level was decreased in the FSGS group, which was consistent with the typical clinical manifestations of common nephrotic syndrome in FSGS group. In addition, light microscopy showed segmental sclerosis of glomeruli, compensatory enlargement of some glomeruli, partial occlusion of capillary lumen, balloon adhesion, increased mesangial matrix, partial tubular atrophy, and renal interstitial fibrosis in FSGS rats. Transmission electron microscopy showed that the morphology of glomerular foot processes disappeared and foot processes were extensively fused, indicating that the FSGS rat model was successfully constructed.

Ferroptosis is an iron-dependent mode of cell death driven by lipid peroxidation which involves complex regulatory mechanisms, mainly the imbalance of antioxidant system, iron metabolism disorder and uncontrolled lipid peroxidation. The antioxidant pathway of ferroptosis is mainly related to the System Xc− -GSH-GPX4 axis. GSH is a necessary co-factor for GPX4 synthesis and exists in the cell in either the oxidized GSSG or the reduced GSH, and a balanced GSH/GSSG ratio is essential for maintaining cell function. GPX4 is able to reduce and detoxify lipid peroxidation, thereby inhibiting ferroptosis, and its depletion is considered a critical step in ferroptosis^18,29^. PUFAs are the main targets of lipid peroxidation and ACSL4 can catalyze the binding of PUFAs to coenzyme A and promote the esterification of PUFAs into phospholipids, which facilitates the downstream cascade of PUFAs, resulting in the generation of phospholipid hydroperoxides (PL-PUFA-OOH). ACSL4 can catalyze the binding of PUFAs to coenzyme A, which is esterified to phospholipids and facilitates the downstream cascade of PUFAs, leading to the formation of phospholipid hydroperoxides (PL-PUFA-OOH), whose accumulation on the cell membrane is a marker and rate-limiting step of ferroptosis^19^. The occurrence of ferroptosis is closely related to the iron accumulation. Iron metabolism involves a complex series of processes, including absorption, storage, utilization, and efflux. The circulating Fe^3+^ enters the cell via transferrin and TFR and is subsequently reduced to Fe^2+^ for transport to the labile iron pool (LIP) in the cytoplasm. This LIP is the source of the Finton reaction. Ferroportin is the only known iron-efflux protein in mammals to date. Ferroportin mediates the entry of Fe^2+^ from the cytoplasm into the blood circulation, a process that is inhibited by hepcidin. Catalyzed by high concentrations of Fe^2+^, phospholipids on the cell membrane are oxidized to highly reactive PL-PUFA-OOH, driving the occurrence of ferroptosis^30^. Thus, accumulation of Fe^2+^ is considered a biomarker of ferroptosis. The results of this study showed that compared with control rats, mitochondria in FSGS rats varied in size, and some mitochondria became smaller in size. And the membrane density of mitochondria increased, and mitochondrial cristae reduced or disappeared. Moreover, there were iron metabolism disorders, such as Fe^2+^ accumulation, increased TFR, hepcidin, decreased ferroportin, accompanied by the accumulation of lipid peroxidation product MDA, and decreased GSH/GSSG in FSGS renal tissue, which was consistent with the characteristics of ferroptosis. In addition, Western blot results showed that GPX4 was depleted and ACSL4 was increased in renal tissue of FSGS rats. The above results suggest that ferroptosis is involved in the development of FSGS.

Multiple cellular metabolic processes can alter cellular sensitivity to ferroptosis by altering intracellular free iron availability because of the iron-dependence of ferroptosis^31^. Autophagy is a degradation pathway that maintains cell renewal and homeostasis under stress stimulation. It can transport intracellular components to lysosomes for degradation^32^. However, both insufficient autophagy and excessive autophagy lead to cell death. Currently, some studies suggest that ferroptosis is an autophagy-dependent cell death that requires selective autophagic forms such as ferritinophagy to execute^33^. In this process, NCOA4, acting as a selective autophagic cargo receptor, directly recognizes and binds to FTH1, a subunit of ferritin, prompting ferritin transport to lysosomes for phagocytosis and degradation, and ultimately increasing the accessibility of free iron. NCOA4-mediated hyperactivation of ferritinophagy increases intracellular iron loading, resulting in the accumulation of ROS and the occurrence of ferroptosis^34^. Fang et al.^35^ reported that compound 9a, a new ferroptosis inhibitor, blocked the binding of NCOA4 to FTH1, thereby reducing the level of intracellular Fe^2+^ and inhibiting ferroptosis. Furthermore, pharmacological inhibition of autophagy and knockdown of NCOA4 significantly alleviated cellular iron overload, thus inhibiting ferroptosis^36^. These results suggest that iron overload is the initial link in ferritinophagy-induced ferroptosis. The results of this study showed that the levels of LC3II/LC3I, NCOA4 and Fe^2+^ in renal tissue were significantly increased, while the expression of FTH1 was significantly decreased in the FSGS rat model. LC3 is a key autophagy marker. During autophagy formation, cytoplasmic LC3I is cleaved and lapidated, transforming into autophagosomal membranous LC3II. There is a dynamic process of generation and degradation between LC3I and LC3II, and LC3II/LC3I ratio is commonly used to assess the level of autophagy^37^. In this study, the LC3II/I ratio was increased in renal tissue of FSGS rats, indicating the formation of autophagosomes and the activation of autophagy. Increased levels of NCOA4 and Fe^2+^ and decreased levels of FTH1 marked the degradation of ferritin and the increase of free iron, thus suggesting the involvement of ferritinophagy in the FSGS rat model, which may be responsible for the induction of ferroptosis in FSGS rats (Supplementary Figures).

**Figure 7:**
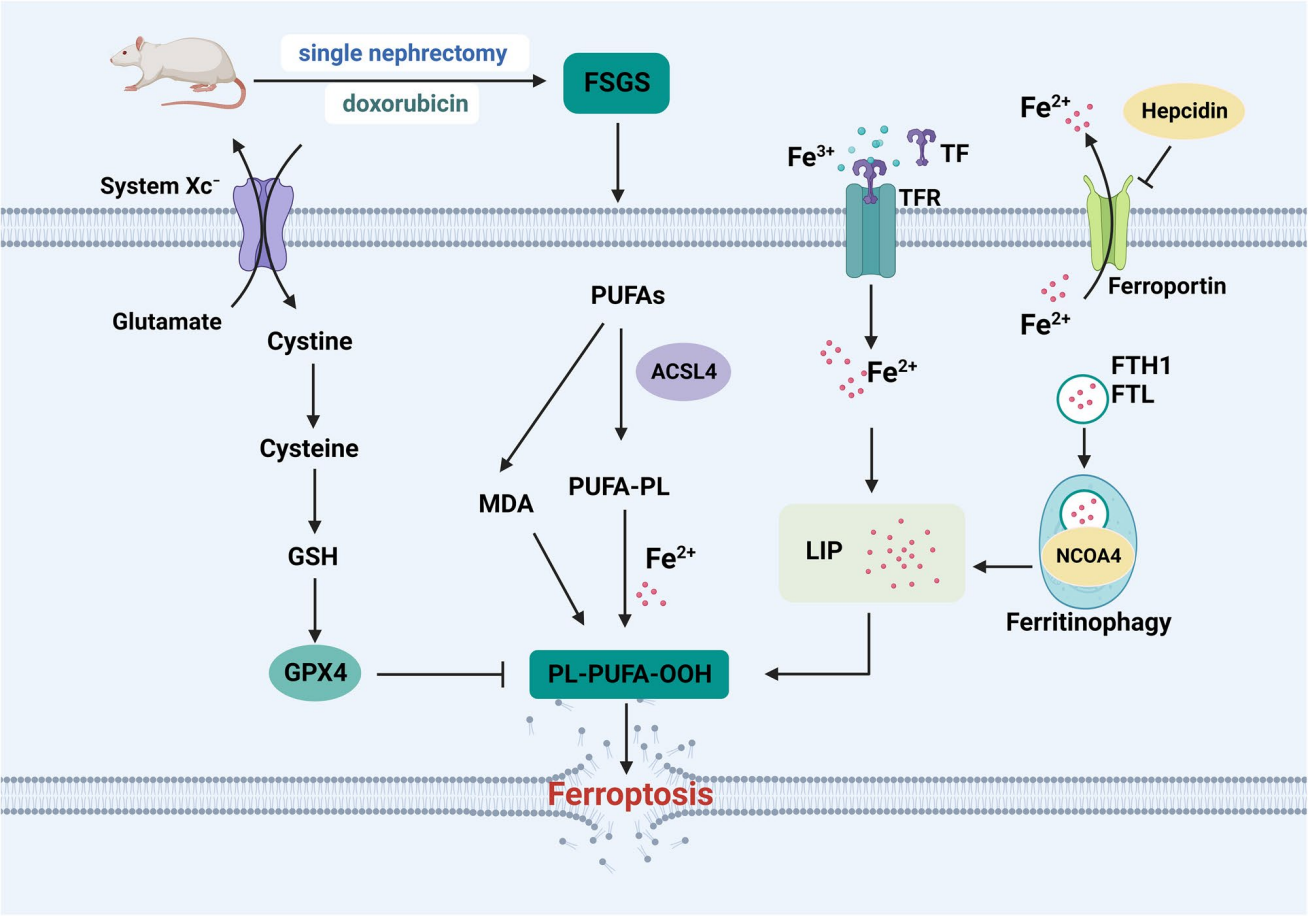
Ferroptosis involved in FSGS in Rats. Fe^3+^ is transported to cells via TF and TFR. Subsequently, Fe^3+^ is reduced to Fe^2+^ and released into LIP. NCOA4 binds to FTH1 and mediate ferritinophagy, increasing the accessibility of Fe^2+^ and inducing ferroptosis. Meanwhile, as a key antioxidant pathway, the system Xc^−^- GSH-GPX4 axis is damaged and promote lipid peroxidation and ferroptosis. PUFAs is converted to PUFA-PL via ACSL4 and generate PL-PUFA-OOH, thus driving ferroptosis. (Abbreviations: FSGS, focal segmental glomerulosclerosis; GSH, glutathione; GPX4, glutathione peroxidase 4; PUFAs, polyunsaturated fatty acids; ACSL4, acyl-CoA synthase long-chain family member 4; PUFA-PL, polyunsaturated fatty acids of phospholipids; MDA, Malondialdehyde; Fe^2+^, ferrous iron; PL-PUFA-OOH, phospholipid hydroperoxides; Fe^3+^, ferric iron; TF, transferrin; TFRC, transferrin receptor; LIP, labile iron pool; FTH1, ferritin heavy chain 1; FTL, ferritin light chain; NCOA4, nuclear receptor coactivator 4).

This study suggests that ferroptosis may be involved in the pathology of FSGS rats, and this process is associated with the activation of ferritinophagy (Fig. 7). Therefore, targeted inhibition of ferritinophagy and ferroptosis is a potential therapeutic strategy for FSGS. However, our study has the following limitations: (1) The exact mechanism of ferroptosis has not been fully revealed, and there is a lack of gold standard specific for the diagnosis of ferroptosis, so the identification of ferroptosis in this study was limited to the observation of mitochondrial morphology, iron metabolism, ACSL4, MDA, GSH/GSSG and GPX4 expression. (2) Preliminary evidence for the existence of ferroptosis in FSGS rats was presented in this study, but inducers and inhibitors related to ferroptosis were not used to further verify the important role of ferroptosis in FSGS rats. In addition, we found activation of ferritinophagy in FSGS rats, speculating that ferritinophagy induced ferroptosis, whereas did not verify a causal relationship between ferritinophagy and ferroptosis by inhibiting pathways involved in ferritinophagy or gene knockout of NCOA4. (3) The role of ferroptosis versus ferritinophagy in FSGS was not verified at the cellular level. These limitations need to be further explored.

In conclusion, ferroptosis was observed in FSGS rats induced by single nephrectomy combined with fractionated tail vein injection of doxorubicin in this study, and this process may be mediated by ferritinophagy. These findings lay the foundation for subsequent studies on the pathogenesis of FSGS and provide reference for expanding its treatment strategies.

### Supplementary Information

Supplementary Figures.

## Data Availability

Data will be made available from the corresponding author on reasonable request.

## Abbreviations

FSGS: Focal segmental glomerulosclerosis
MDA: Malondialdehyde
GSH: Glutathione
GPX4: Glutathione peroxidase 4
GSSG: Glutathione oxidized
ACSL4: Acyl-CoA synthase long-chain family member 4
NCOA4: Nuclear receptor coactivator 4
FTH1: Ferritin Heavy Chain 1
ESRD: End-stage renal disease
ROS: Reactive oxygen species
PUFAs: Polyunsaturated fatty acids
AKI: Acute kidney injury
CKD: Chronic kidney disease
DKD: Diabetic nephropathy
BUN: Blood urea nitrogen
Scr: Serum creatinine
TC: Total cholesterol
TG: Triglyceride
ALB: Albumin
HE: Hematoxylin–eosin
GBM: Glomerular basement membrane
PL-PUFA-OOH: Phospholipid hydroperoxides
TF: Transferrin receptor
LIP: Labile iron pool
