# Supplementary material for: Ferroptosis is involved in focal segmental glomerulosclerosis in rats
Source: Sci Rep. 2023 Dec 14;13:22250. doi: 10.1038/s41598-023-49697-8 (PMC10721625; doi:10.1038/s41598-023-49697-8)

**Ferroptosis is involved in focal segmental glomerulosclerosis in rats**

Yue Shi^a^, Xiujie Shi^a^, Mingming Zhao^a^, Yifan Zhang^a^, Qi Zhang^a^, Jing Liu^a^, Hangyu Duan^a^, Bin Yang^b*^, Yu Zhang^a*^

^a^Department of Nephrology, Xiyuan Hospital, China Academy of Chinese Medical Sciences, Beijing 100091, China

^b^Department of Pathology, Xiyuan Hospital, China Academy of Chinese Medical Sciences, Beijing 100091, China

*Corresponding author

Bin Yang: No. 1, Xiyuan Playground, Haidian District, Beijing; e-mail address: yangbin555@126.com

Yu Zhang: No. 1, Xiyuan Playground, Haidian District, Beijing; e-mail address: zhangyu8225@126.com

Figure 4C Gel images of hepcidin, ferroportin and TFR










Figure 5E Gel images of GPX4 and ACSL4


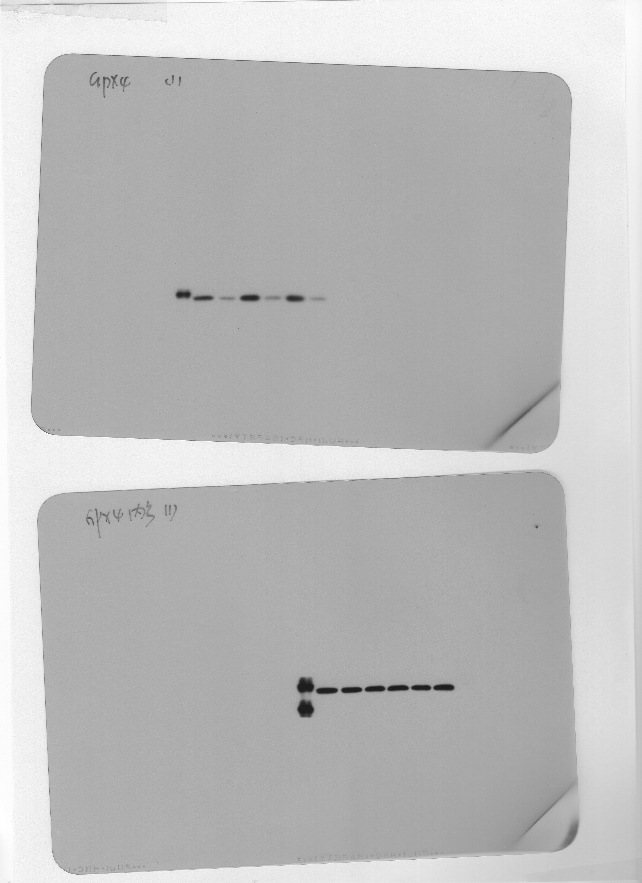




Figure 6A Gel images of LC3II/LC3I, FTH1 and NCOA4







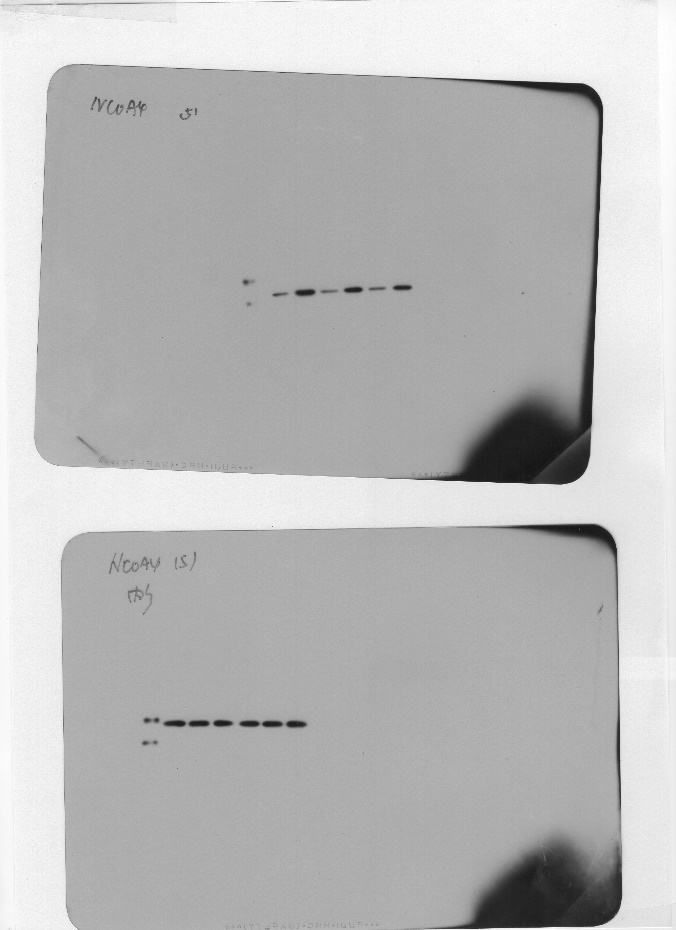

Supplement: Supplementary file 1 — Supplementary Figures. [file 41598_2023_49697_MOESM1_ESM.docx]
